# Retinol-binding protein 4 in cardiovascular diseases: mechanisms and therapeutic perspectives

**DOI:** 10.3389/fcvm.2026.1703840

**Published:** 2026-01-29

**Authors:** Jiefang Chen, Gaijie Chen, Xiaojing Xu, Jiewen Zhang, Feng Liu

**Affiliations:** 1 Department of Neurology, Henan Provincial People’s Hospital, People’s Hospital of Zhengzhou University, Zhengzhou, China; 2 Health Management Center, Henan Provincial People’s Hospital, People’s Hospital of Zhengzhou University, Zhengzhou, China; 3 Department of Respiratory and Critical Care Medicine, Henan Provincial People’s Hospital, People’s Hospital of Zhengzhou University, Zhengzhou, China; 4 Department of Nephrology, Henan Provincial People’s Hospital, People’s Hospital of Zhengzhou University, Zhengzhou, China

**Keywords:** biomarker, CVDs, RBP4, risk factor, therapeutic target

## Abstract

Cardiovascular diseases (CVDs) continue to be the leading cause of mortality worldwide, highlighting the need for enhanced diagnostic tools to enable early intervention. However, the complexity of these diseases poses significant challenges to their diagnosis and management. Therefore, a deeper understanding of CVD mechanisms and the development of novel diagnostic and therapeutic strategies are of critical importance. Retinol-binding protein 4 (RBP4), a member of the lipocalin family, is mainly secreted by the liver and adipose tissue and is widely recognized for its role in transporting retinol (vitamin A). Beyond functioning as a selective retinol carrier, growing evidence suggests that RBP4 is intricately involved in the pathogenesis of CVDs and their associated risk factors. Although numerous studies have established a link between RBP4 and the onset and progression of CVDs, the underlying mechanisms remain incompletely elucidated. This review summarizes the biological characteristics and multifunctional roles of RBP4 in CVD pathophysiology, examines its potential as a biomarker for diagnosis and prognosis, and explores its implications for developing new strategies to prevent and treat cardiovascular disorders.

## 1 Introduction

Cardiovascular diseases (CVDs) remain the foremost cause of global morbidity and mortality (1). Among these, coronary artery disease (CAD) and stroke represent the main contributors to this burden (2). Ischemic heart disease (IHD), most commonly resulting from coronary artery stenosis or occlusion due to atherosclerosis (AS) or thrombosis, is a major manifestation of CVDs (3). Stroke, which encompasses both ischemic and hemorrhagic types, is predominantly ischemic in nature, accounting for approximately 85% of all cases (4). Established traditional risk factors for CVDs include hypertension, dyslipidemia, diabetes, and obesity (5). Nevertheless, a notable proportion of patients experiencing cardiovascular events do not exhibit any of these conventional risk factors (6, 7), indicating that other underlying mechanisms may influence the onset and progression of CVDs. Therefore, exploring personalized risk factors to accurately identify individuals at high risk of cardiovascular events is an important challenge in current CVDs management.

Retinol binding protein 4 (RBP4), first isolated from human serum by Kahn et al. in 1968, belongs to the lipocalin family. It is primarily secreted by the liver and adipose tissue, with a molecular weight of approximately 21 kDa. The primary role of RBP4 is to transport retinol (vitamin A) from the liver to peripheral tissues, where it is metabolized into retinoic acid (8, 9). Beyond the liver and adipose tissue, RBP4 mRNA is also detectable in the kidney, brain, and lungs (10). RBP4 exists in two forms: apo-RBP4, which is unbound to retinol, and holo-RBP4, which binds to retinol and also associates with transthyretin (TTR) in plasma to prevent renal filtration and excretion (11). Notably, urinary RBP4 has been proposed as a potential biomarker for assessing kidney function (12, 13). The biological actions of RBP4 are mediated mainly through two receptors: Toll-like receptor 4 (TLR4) and the cell-membrane receptor STRA6 (stimulated by retinoic acid 6). Binding to TLR4 activates the c-Jun N-terminal kinase (JNK) pathway, whereas interaction with STRA6 triggers the Janus kinase 2/signal transducer and activator of transcription 5 (JAK2/STAT5) signaling cascade (14–18). Circulating RBP4 is predominantly derived from hepatocytes, with a minor contribution from adipocytes and other cell types (19). In humans, the physiological circulating concentration of RBP4 typically ranges from 2 to 3 µmol/L, whereas in mice it is generally between 0 and 1 µmol/L (20). Elevated levels of RBP4 have clinical significance: serum concentrations exceeding 55 µg/mL are associated with a nearly twofold increase (1.97-fold) in the risk of developing type 2 diabetes mellitus (T2DM) (21). Similarly, epidemiological evidence indicates that a 25% rise in circulating RBP4 corresponds to an approximately 2.5-fold higher risk of cardiovascular diseases (13).

Initially, research primarily linked RBP4 to obesity, insulin resistance (IR), and type 2 diabetes mellitus (T2DM) (22). In contrast, numerous subsequent studies have associated elevated serum RBP4 levels with various CVDs, including CAD (23), heart failure (24), and ischemic stroke (IS) (25–28). Furthermore, increased RBP4 levels are correlated with several established risk factors for CVDs, such as inflammation (16, 29, 30), hypertension (25, 31), dyslipidemia (28, 32, 33), and AS (34–36), all of which contribute to the initiation and progression of cardiovascular pathology. Additionally, elevated RBP4 expression in epicardial adipose tissue has been reported in association with left ventricular diastolic dysfunction (37). Notably, critically ill patients present with significantly lower serum RBP4 levels compared to healthy controls, and low RBP4 levels have been identified as an unfavorable predictor of short-term mortality in the intensive care unit (ICU) (38). In this review, we synthesize and update recent evidence on the multifaceted role of RBP4 in CVDs and their risk factors, offering insights into its potential translational and clinical applications.

## 2 RBP4 structure and function

### 2.1 RBP4 structural characteristics

RBP4 is a single-polypeptide protein composed of 201 amino acid residues, with a molecular weight of approximately 21 kDa (39). Its core structure consists of a characteristic β-barrel, which binds specifically to one molecule of all-trans retinol—the active form of vitamin A. This binding enables retinol to remain soluble in aqueous environments and facilitates its transport in the bloodstream. The overall RBP4 molecule contains an N-terminal ring, an α-helix, and a C-terminal ring, which collectively maintain protein stability and function. In circulation, RBP4 forms a 1:1:1 complex with retinol and TTR, which enhances the stability and solubility of retinol while preventing its oxidation and toxic accumulation (10, 40). These structural properties establish RBP4 as an essential mediator of vitamin A transport *in vivo* and link it closely to metabolic regulation and disease pathophysiology.

### 2.2 RBP4 ligand interactions

The interaction between RBP4 and ligands mainly involves the following aspects (Table 1):

**Table 1:** Known ligands and interaction partners of RBP4.

Ligand	Binding site	Affinity	Experimental Validation (*in vitro*/*in vivo*)	Function	References
Retinol	*β*-barrel core	High affinity	Both *in vitro* & *in vivo*	Delivers retinol to tissues (e.g., retina, liver).	(10)
STRA6	Unknown	High affinity	*In vitro*	promote retinol entry into the cell regulate intracellular signaling pathways	(42, 43)
RBPR2	Unknown	High affinity	*In vitro*	participate in retinol uptake affect insulin resistance	(44)
TLRs	Unknown	Unknown	Both *in vitro* & *in vivo* (animal)	induce the release of inflammatory factors induce insulin resistance regulate intracellular signaling pathways	(45)
RAR/ RXR	No (indirect interaction)	No (indirect interaction)	Both *in vitro* & *in vivo* (animal)	regulating gene transcription promoting lipid synthesis	(47–49)

**Binding to Retinol**: (1) The primary function of RBP4 is to specifically bind and transport retinol (the active form of vitamin A) from the liver to target tissues. Before leaving hepatocytes, RBP4 binds retinol to form holo-RBP4, which then assembles in the endoplasmic reticulum into a 1:1:1 complex with TTR. This complex prevents renal filtration and degradation of RBP4 during circulation (41). Retinol binding to RBP4 is reversible; upon reaching target cells, the vitamin is internalized via membrane receptors such as STRA6 (also known as RBP4 receptor 1, RBPR2), thereby completing vitamin A uptake and metabolic regulation (10).

**Interaction with the STRA6 Receptor:** When the circulating RBP4-retinol-TTR complex reaches target cell, TTR dissociates to allow RBP4-retinol to bind STRA6, facilitating retinol entry into the cell (42). Beyond its transport role, STRA6 also functions as a signaling receptor (43). Its activation can modulate intracellular pathways, including the JAK/STAT cascade, thereby influencing insulin sensitivity and lipid metabolism.

**Interaction with the RBPR2 Receptor:** Identified in 2013, RBPR2 (stimulated by retinoic acid 6-like, STRA6L) shares about 20% homology with STRA6. It is predominantly expressed in the liver, intestine, and adipose tissue under obese conditions. RBPR2 participates in retinol uptake and has been implicated in the regulation of insulin resistance (44).

**Interaction with TLR Receptors:** In adipose tissue, RBP4 can bind to and activate Toll-like receptors—specifically TLR2 and the TLR4/MD-2 complex—on the surface of macrophages. This triggers the release of inflammatory cytokines, such as TNF-α and IL-1β, and contributes to the pathogenesis of insulin resistance and metabolic dysregulation (45).

**Indirect Interaction with Nuclear Receptors:** Under pathological conditions, elevated circulating RBP4 enhances retinol delivery to tissues. In the liver, retinol is metabolized into retinoic acid (RA) isomers, which act as ligands for nuclear receptors including the retinoic acid receptor (RAR) and retinoid X receptor (RXR) (46). Activation of these receptors regulates gene transcription and can promote processes such as lipid synthesis (47–49).

In summary, RBP4 participates in various physiological and pathological processes including vitamin A metabolism, insulin resistance, and inflammatory response through interactions with ligands such as retinol, receptors, and nuclear receptors.

### 2.3 RBP4-related signal transduction

**Figure 1:**
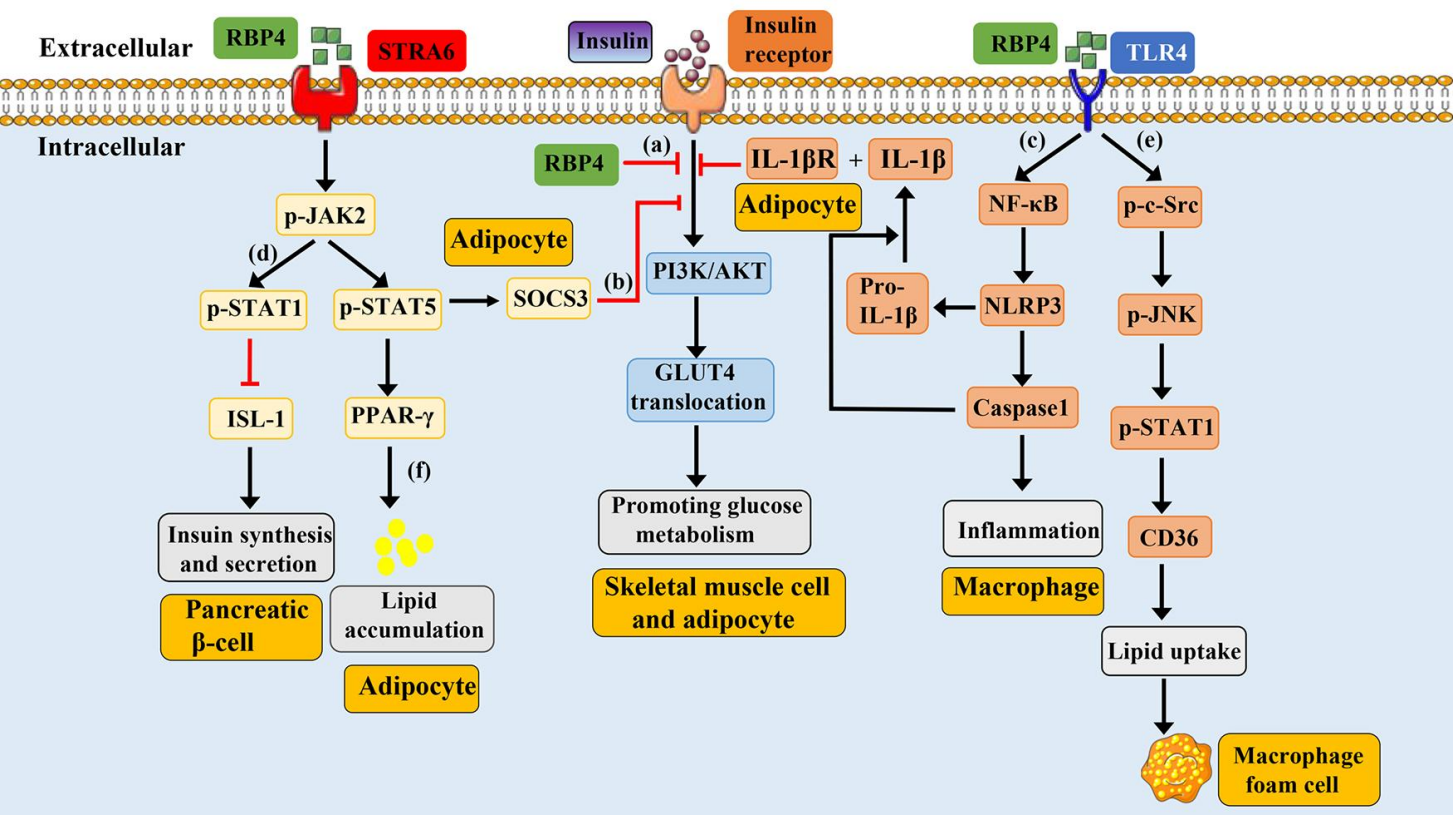
Schematic representation of RBP4-mediated signaling pathways. Under physiological conditions, the binding of insulin-to-insulin receptors promotes the activation of PI3K/AKT, leading to the translocation of GLUT4 and promoting glucose metabolism. **(a)**: Overexpression of mouse RBP4 or injection of purified human RBP4 recombinant protein into mice can inhibit insulin induced activation of PI3K/AKT. **(b)**: Circulating RBP4 can recruit and activate JAK2 by binding to the adipocyte surface receptor STRA6, thereby promoting phosphorylation of STAT5. Subsequently, phosphorylated STAT5 enters the nucleus, causing the expression of target genes SOCS3 and PPAR-γ, which respectively inhibit the insulin signaling pathway and promote lipid aggregation. **(c)**: RBP4 can promote the phosphorylation of NF-κB by binding to TLR4 receptors on the surface of macrophages, thereby promoting the formation of NLRP3 inflammasome, leading to the synthesis and release of IL-1β. IL-1β binds to IL-1βR on the surface of adipocytes and induce the inhibition of the insulin signaling pathway. **(d)**: RBP4 binds to the pancreatic cell surface receptor STRA6, causing phosphorylation of JAK, which in turn leads to phosphorylation of STAT1. Phosphorylated STAT1 enters the nucleus and inhibits the expression of ISL1 gene, resulting in reduced insulin synthesis and secretion. **(e)**: RBP4 binds to the TLR4 receptor on the surface of macrophages, causing phosphorylation of c-src and JNK. The phosphorylation of JNK can promote the phosphorylation and nuclear translocation of STAT1. The phosphorylated STAT1 binds to the promoter of CD36 to promote its expression, which then leads to increased cholesterol uptake and the formation of foam cells. **(f)**: RBP4 can recruit and activate JAK2 by binding to the adipocyte surface receptor STRA6, thereby promoting phosphorylation of STAT5. Subsequently, phosphorylated STAT5 enters the nucleus, causing the expression of target genes PPAR-γ, leading to lipid aggregation. GLUT4, glucose transport protein 4; JAK2, Janus kinase 2; STAT5, signal transducer and activator of transcription 5; SOCS3, cytokine signaling 3; PI3K/AKT, phosphatidylinositol 3-kinase/protein kinase B; TLR4, toll-like receptor 4; NLRP3, nucleotide-binding domain and leucine-rich repeat containing protein 3; IL-1β, interleukin-1 beta; NFκB, nuclear factor kappa B; STRA6, stimulated by retinoic acid 6; STAT1, signal transducer and activator of transcription 1; ISL-1, insulin gene enhancer binding protein 1; JNK, jun N-terminal kinase; PPARγ, peroxisome proliferator-activated receptor gamma.

The signaling pathways associated with RBP4 and their potential physiological effects are summarized as follows (Figure 1).

**Insulin Resistance-Related Signaling:** RBP4 directly inhibits the phosphatidylinositol 3-kinase/protein kinase B (PI3K/AKT) pathway in skeletal muscle cells, reduces glucose transport protein 4 (GLUT4) translocation, and impairs glucose uptake (9). Additionally, by binding to the STRA6 receptor on adipocytes, RBP4 activates the JAK2/STAT5/SOCS3 cascade, contributing to insulin resistance (43). Furthermore, through the TLR4/NF-κB/NLRP3 pathway, RBP4 promotes the release of pro-inflammatory cytokines such as TNF-α and IL-1β from macrophages in adipose tissue, thereby inducing insulin resistance in adipocytes (45).

**Insulin Synthesis-Related Signaling:** In pancreatic islet cells, RBP4 binding to STRA6 activates the JAK2/STAT1 pathway, which suppresses transcription factors involved in insulin synthesis (e.g., ISL-1), ultimately inhibiting insulin production (50).

**Inflammatory Signaling:** RBP4 can enhance the expression of pro-inflammatory markers in CD206^+^ macrophages via a JNK-dependent mechanism and promote the polarization of CD4^+^ T cells toward a TH1 phenotype, thereby triggering adipose tissue inflammation. This inflammatory response may further activate TLR4 and JNK signaling, increase the release of pro-inflammatory cytokines, and exacerbate insulin resistance and metabolic dysregulation (51).

**Retinol Metabolism and Transport:** RBP4 binds retinol to form a complex that is stabilized in circulation by association with TTR, ensuring efficient delivery to target tissues (41). At target cells, RBP4 interacts with the STRA6 receptor to mediate retinol endocytosis and intracellular release, thereby participating in retinol metabolism and physiological regulation. Retinol can further activate nuclear receptors such as RAR and RXR, modulating gene expression (47, 48).

**CVDs-Related signaling:** Elevated RBP4 levels are linked to CVDs including AS, CAD, and stroke (28, 52). Mechanistically, RBP4 may activate TLR4/JNK and TLR4/NF-κB/NLRP3 pathways, promoting inflammatory cell activation and cytokine release, which drives vascular inflammation (15, 45, 51). It may also enhance endothelial oxidative stress (53), facilitate foam cell formation (36), and induce lipid accumulation (43), collectively accelerating the progression of atherosclerotic plaques.

Dysregulation of these RBP4-associated signaling pathways is closely implicated in the pathogenesis of various diseases, such as diabetes, AS, stroke, and CAD. This underscores the potential of RBP4 as a therapeutic target for related disorders.

## 3 RBP4 in CVDs

CVDs encompass a range of disorders, among which CAD and stroke represent leading global causes of disability and mortality. AS underlies the majority of these conditions (54). Accordingly, this section focuses on the associations between RBP4 and CAD, stroke, and AS.

### 3.1 RBP4 in CAD

CAD, also referred to as coronary heart disease (CHD) or IHD, is the most frequent cause of myocardial infarction and imposes a substantial burden on individuals and society (55). In 2013, Sun et al. reported that elevated circulating RBP4 levels were associated with a threefold increase in CHD risk (56). Subsequent work by Li et al. indicated that serum RBP4 correlates with cardiovascular risk factors and may serve as a marker for CAD (57). Similarly, Dong et al. compared patients with and without carotid atherosclerotic plaques, as well as with and without CHD, and found significantly higher serum RBP4 levels in both plaque-positive and CHD groups. They proposed RBP4 as an independent risk factor for carotid plaque formation and CHD (58), a conclusion consistent with that of Lambadiari et al. (59). Another study by Liu et al. also observed higher serum RBP4 levels in CAD patients compared to controls (60). Together, these studies support the potential of RBP4 as a predictive and diagnostic marker for CAD. Beyond diagnosis, RBP4 has been linked to CAD complexity and severity (23, 59, 61, 62). Moreover, increased circulating RBP4 is associated with a higher risk of major adverse cardiovascular events (MACEs)—including acute coronary syndrome (ACS), heart failure, stroke, peripheral vascular disease, and cardiovascular death—in patients with stable CAD (63). A prospective study further reported that in patients with ACS, higher serum RBP4 at onset predicted a greater risk of MACEs during follow-up, suggesting its utility as a prognostic indicator in ACS (64) (Table 2). These findings collectively highlight RBP4 as a promising biomarker for the diagnosis and prognosis of CHD. Early monitoring and management of serum RBP4 levels could therefore aid in the prevention, timely diagnosis, and treatment of CHD.

**Table 2:** RBP4 and CAD.

Date	Group A	Group B	Main finding	Reference
In 2013	468 individuals with CAD	472 matched controls	High RBP4 levels were associated with a 3-fold increased risk of incident CHD in women.	(55)
In 2014	30 with CAD and 30 with CAD and hyperinsulinemia (CAD/HIns)	29 healthy subjects	The serum RBP4 concentrations were significantly higher in the CAD/HIns than in the CAD and control groups and associated with cardiovascular risk factors.	(56)
In 2015	50 cases with CHD	160 cases without CHD	The serum RBP4 level was significantly higher in CHD patients than in non-CHD patients (45.94 ± 7.85 mg/L vs. 42.21 ± 6.42 mg/L).	(57)
In 2014	305 individuals with CAD	91 individuals without CAD	Serum RBP4 levels were significantly elevated in patients with CAD compared to non-CAD patients (39.29 ± 11.72 mg/L vs. 24.83 ± 11.27 mg/L).	(58)
In 2019	CAD group (*n* = 180)	Control group (*n* = 79)	The serum RBP4 level was significantly higher in CAD group than in control group (5.79 ng/mL vs. 3.6 ng/mL).	(59)
In 2021	55 patients with presenting acute coronary syndrome (ACS)	43 control subjects	Serum RBP4 levels were significantly higher in patients with ACS compared to the without ACS (68.40 ± 47.94 mg/L vs. 49.46 ± 13.64 mg/L).	(61)

### 3.2 RBP4 in IS

IS is characterized by a sudden disruption of cerebral blood flow due to thrombosis or embolism, and remains a leading cause of mortality and morbidity worldwide (65, 66). Elevated serum RBP4 levels have been associated with an increased risk of cerebrovascular disease in elderly men (25). Consistently, Wang et al. reported significantly higher serum RBP4 levels in elderly patients with cerebral infarction (67). In a study by Sasaki et al., plasma RBP4 was found to be elevated in 58 IS patients compared with 53 healthy controls when measured on the morning after admission (28). Chao et al. further confirmed these findings and demonstrated that RBP4 levels correlated with both infarct size on MRI and stroke severity assessed by the National Institutes of Health Stroke Scale (NIHSS) on admission (26). Beyond severity, higher plasma RBP4 has also been linked to poorer 3-month functional outcomes after stroke (27, 52). Notably, RBP4 levels are reported to be higher in IS than in intracerebral hemorrhage (ICH), suggesting its potential utility in differentiating between these stroke subtypes (68) (Table 3). Regarding the underlying mechanisms, another study indicated that the RBP4/ Lipoprotein-associated phospholipase A2 (Lp-PLA2)/Netrin-1 pathway is involved in the onset and progression of diabetic nephropathy complicated with silent cerebral infarction and cognitive decline (69). Lp-PLA2 promotes inflammatory responses, contributes to the initiation and progression of atherosclerosis, and may facilitate plaque rupture (70–72). Netrin-1, originally identified as an axonal guidance molecule, also exerts anti-inflammatory, anti-apoptotic, and vascular protective effects (73, 74). It is hypothesized that RBP4 may upregulate Lp-PLA2 and downregulate netrin-1, thereby amplifying vascular inflammation and impairing endogenous protective responses, which could collectively promote AS and increase susceptibility to cerebral infarction. While this represents one plausible mechanistic link between RBP4 and IS, the exact molecular pathways remain incompletely understood and warrant further investigation through cellular and animal studies. In summary, RBP4 shows promise as a diagnostic biomarker for IS and a predictor of stroke prognosis. Furthermore, RBP4 antagonists may hold therapeutic potential as neuroprotective agents, and early intervention targeting RBP4 could potentially improve outcomes in stroke patients.

**Table 3:** RBP4 and IS.

Date	Group A	Group B	Main findings	Reference
In 2019	323 patients with AIS	323 healthy people	The serum RBP4 levels were significantly (*P* < 0.001) higher in patients with AIS than in those normal cases [28.9 (IQR, 17.3–39.6) μg/mL vs. 23.7 (14.6–32.3) μg/mL]. In patients with a minor stroke (NIHSS < 6, *n* = 116), the median RBP4 level was lower than that in patients with moderate-to-high clinical severity [19.4 (IQR, 12.4–28.1) μg/mL vs. 33.7 (25.4–42.2) μg/mL.	(26)
In 2018	299 first-ever AIS	150 age and gender-matched healthy volunteers	The serum levels of RBP4 were significantly higher in stroke patients as compared with normal cases [27.3 (IQR: 18.6–36.4) μg/mL compared with 17.6 (IQR: 11.8–23.5) μg/mL; *P* < 0.001]. There was a correlation between levels of RBP4 and NIHSS score [*r*(Spearman) = 0.437, *P* < 0.0001]. There was a positive correlation between levels of RBP4 and the infarct volume (*r* = 0.316, *P* < 0.001.) Significantly higher RBP4 values were found in poor 3-month functional outcomes rather than good outcomes' patients [37.2 (IQR: 25.8–46.2) μg/mL compared with 24.8 (IQR:15.7–31.8) μg/mL; *P* < 0.001].	(27)
In 2010	58 subjects with cerebral infarction	53 control subjects	Plasma RBP4 was 16.4 ± 2.8 μg/mL in the subjects with cerebral infarction, a value significantly greater than that of 10.1 ± 1.2 μg/mL in the controls.	(28)
In 2022	136 patients with cerebral infarction	40 age- and sex-matched control participants	The serum levels of RBP4 were significantly elevated in elderly patients with cerebral infarction than in control participants. The levels of RBP4, 8-iso-PGF2α, and IMT were higher in the unstable plaque group than that in the stable plaque group. The serum level of RBP4 positively correlated with the 8-iso-PGF2α level, IMT, and carotid plaque area.	(66)

### 3.3 RBP4 in AS

AS remains the primary pathological basis of CVDs worldwide and is recognized as a chronic inflammatory condition. Characterized by high incidence, disability, and mortality rates, AS poses a serious threat to human life and health (75–78). CVDs resulting from AS are collectively termed atherosclerotic cardiovascular disease (ASCVD), which primarily includes IHD and IS (79). Elevated levels of RBP4 have been reported to correlate positively with carotid intima–media thickness (IMT) (35). IMT is not only one of the most well-established markers of AS but also an important predictor of CVD risk, holding significant diagnostic and monitoring value in clinical practice (80). Furthermore, one study indicated that circulating RBP4 levels are higher in patients with carotid AS compared to healthy controls (34). Wan et al. demonstrated that RBP4 participates in the initiation and progression of AS in diabetic rats via the Janus kinase 2/signal transducer and activator of transcription 3 (JAK2/STAT3) signaling pathway (81). These findings collectively suggest that RBP4 is involved in the pathogenesis and progression of AS. However, the potential mechanisms of RBP4 in atherosclerotic development remain insufficiently studied. Current treatment for AS largely relies on lipid-lowering statins. Nevertheless, the efficacy of statins is limited, with only about 21% of treatments associated with reduced plaque progression (82). In this context, we summarize the role of RBP4 in endothelial cells (ECs), vascular smooth muscle cells (VSMCs), and macrophages, aiming to provide new insights for the diagnosis and treatment of AS.

#### 3.3.1 RBP4 in ECs

ECs serve as the primary interface susceptible to damage across various blood vessel types. ECs dysfunction is widely regarded as the initial step in the development of AS (83). In 2008, Park et al. conducted a study involving 50 patients with type 2 diabetes mellitus (T2DM), measuring serum levels of RBP4, soluble intracellular adhesion molecule-1 (sICAM-1), and soluble E-selectin (sE-selectin). The latter two markers are elevated upon ECs injury (84, 85), and their results demonstrated a positive correlation between RBP4 and both sICAM-1 and sE-selectin (86). Another study further supports the association between RBP4 and ECs dysfunction (87), consistent with the findings of Park et al. Similarly, *in vitro* experiments indicate that RBP4 promotes the secretion of various proinflammatory factors from ECs, including vascular cell adhesion molecule-1 (VCAM-1), sICAM-1, sE-selectin, monocyte chemoattractant protein-1 (MCP-1), and interleukin-6 (IL-6), via activation of NADPH oxidase and NF-κB. Notably, both apo-RBP4 (retinol-free) and holo-RBP4 (retinol-bound) exerted comparable pro-inflammatory effects, suggesting that the activity of RBP4 is independent of retinol (16). Moreover, multiple studies have reported positive correlations between RBP4 and oxidative stress biomarkers such as urinary 8-isoprostane (53), 8-iso-prostaglandin F2*α* (8-isoPGF2α) (88), 13-(S)-hydroxy octadecadienoic acid (88), and malondialdehyde (87), along with a negative correlation with the antioxidant glutathione (53). Given that cellular oxidative stress can directly trigger ECs inflammation (89, 90), RBP4 may not only directly stimulate the release of inflammatory factors from ECs but also promote ECs inflammation through oxidative stress pathways. However, further research is needed to clarify the precise mechanisms involved. Beyond its pro-inflammatory role, RBP4 has also been shown to promote ECs apoptosis by inducing mitochondrial dysfunction and vascular oxidative damage, mediated via suppression of the PI3K/AKT signaling pathway, both *in vivo* and *in vitro* (91). Collectively, these findings suggest that RBP4 likely contributes to the initiation and progression of AS by inducing ECs inflammation. Based on the evidence summarized above, serum RBP4 levels may reflect ECs injury and serve as an independent marker of ECs dysfunction. This could facilitate early detection of endothelial damage and enable timely interventions to prevent the onset of vascular diseases.

#### 3.3.2 RBP4 in VSMC

VSMCs are integral components of the vascular wall and participate in nearly the entire process of AS. The proliferation, migration, and phenotypic transformation of VSMCs significantly contribute to the progression of AS pathogenesis (92–94). Both *in vivo* and *in vitro* studies have demonstrated that RBP4 promotes VSMC proliferation and migration through the JAK2/STAT3 signaling pathway, an effect that can be inhibited by vitamin D (95). Furthermore, Fei Li et al. reported that high insulin levels promote VSMC proliferation via activation of the mitogen-activated protein kinase (MAPK) signaling pathway, and RBP4 enhances this effect (96, 97). Under physiological conditions, VSMCs maintain a contractile phenotype, expressing various contractile proteins essential for vascular elasticity and integrity. Under pathological conditions, however, VSMCs can undergo a transition to a synthetic/secretory phenotype. This shift leads to increased secretion of extracellular matrix components, lipid accumulation, and ultimately promotes both the progression of AS and plaque instability (98). Importantly, phenotypic transformation of VSMCs is considered an initial step preceding their proliferation and migration (99). In this context, Wan Zhou et al. showed that RBP4 can promote the phenotypic switch of VSMCs by activating the RhoA (ras homolog family member A)/ROCK1 (Rho associated coiled-coil containing protein kinases) signaling pathway (100). Although research on RBP4 and VSMCs remains relatively limited, current evidence indicates that RBP4 likely promotes AS by stimulating VSMC proliferation, migration, and phenotypic switching. The specific molecular mechanisms underlying these effects, however, are not yet fully understood and warrant further investigation.

#### 3.3.3 RBP4 in macrophages

Macrophages play crucial roles in the initiation and progression of aAS by phagocytosing oxidized low-density lipoprotein (Ox-LDL) to form foam cells, participating in inflammatory responses, altering their phenotypes, and secreting a variety of cytokines (101–103). In 2008, M. Broch et al. first reported that RBP4 is expressed in differentiated macrophages but not in undifferentiated monocytes, and its expression can be modulated by inflammatory stimuli (104). This finding marked an important milestone and opened new avenues for investigating the role of macrophages in AS. Studies have demonstrated that RBP4 promotes atherosclerotic progression by enhancing macrophage foam cell formation. This is achieved through activation of the c-Src–JNK–STAT1 pathway, which upregulates CD36 expression and increases cellular cholesterol uptake (36). Beyond facilitating foam cell formation, RBP4 is also implicated in macrophage-driven inflammation. Research by Julie Norseen et al. revealed that RBP4 can induce the secretion of pro-inflammatory cytokines from macrophages via the TLR4/JNK signaling pathway, an effect that is independent of retinol (15). Thus, within macrophages, RBP4 promotes AS through two key mechanisms: augmenting foam cell formation and amplifying inflammatory activation.

In summary, RBP4 contributes to AS through a tripartite mechanism: (a) inducing ECs inflammation and dysfunction, (b) stimulating VSMC proliferation, migration, and phenotypic switching, and (c) enhancing macrophage foam cell formation and pro-inflammatory responses within plaques. Although research on the relationship between RBP4 and AS continues to evolve, current evidence strongly supports its role in facilitating AS onset and progression. These findings highlight the potential of RBP4 as a dual-purpose diagnostic marker and therapeutic target, which may inform novel clinical strategies. Nevertheless, the precise molecular mechanisms and downstream signaling pathways mediated by RBP4 in AS remain incompletely understood. Future studies should therefore prioritize elucidating these mechanisms to fully clarify the pathogenic role of RBP4 in AS.

## 4 RBP4 and risk factors for CVDs

### 4.1 RBP4 and obesity, IR and T2DM

Elevated circulating levels of RBP4 have been reported in obese individuals compared with lean subjects, paralleling an increased prevalence of IR—a core pathological feature of T2DM (22, 105–107). One study further indicated that when circulating RBP4 exceeds 55 μg/mL, the risk of T2DM rises by 1.97-fold (21). Interestingly, Graham et al. demonstrated that a 4-week exercise intervention can lower RBP4 levels and improve IR (22). IR denotes diminished tissue sensitivity to insulin, resulting in reduced glucose uptake and utilization and consequent hyperglycemia (107). Notably, IR is an independent risk factor for CVD and early mortality even in the absence of diabetes (108, 109). It can promote endothelial dysfunction by inducing oxidative stress, stimulating cytokine production, and disrupting the renin–angiotensin–aldosterone system (110). Moreover, IR accelerates the progression of atherosclerotic plaques toward a vulnerable phenotype (111), whose rupture may trigger thrombosis and lead to stroke, myocardial infarction, or other acute vascular events (112). Compensatory hyperinsulinemia often arises because of reduced insulin sensitivity (113–116). Elevated insulin levels can drive AS through multiple mechanisms, including stimulation of very-low-density lipoprotein (VLDL) synthesis and secretion (117, 118), promotion of VSMC proliferation and growth (119, 120), activation of inflammation-related genes (121, 122), increased collagen synthesis (123, 124), and enhanced low-density lipoprotein (LDL) transport into smooth muscle cells (SMCs) (125, 126). In rats, insulin infusion under euglycemic conditions induces hypertension—another independent CVD risk factor (127, 128). Clinically, hyperinsulinemia is also associated with elevated risk and progression of several cancers, including those of the pancreas, breast, and colon (129).

Skeletal muscle and adipose tissue are primary sites of insulin-mediated glucose metabolism. Physiologically, insulin binding to its receptor activates the PI3K/AKT pathway, leading to GLUT4 translocation and enhanced glucose uptake. Under pathological conditions, however, key proteins in this pathway are suppressed, resulting in IR (130). Accumulating evidence links RBP4 to IR (9, 61, 131). Qin Yang et al. showed that either transgenic overexpression of RBP4 or intraperitoneal injection of recombinant human RBP4 in mice reduced insulin-stimulated PI3K activity in muscle by 30% and 34%, respectively, inducing IR (9). Beyond TLR4, the cell-surface receptor STRA6 also mediates RBP4-induced insulin resistance. Daniel C. et al. reported that holo-RBP4 binding to STRA6 recruits and activates JAK2, leading to STAT5 phosphorylation, nuclear translocation, and subsequent upregulation of cytokine signaling 3 (SOCS3) and peroxisome proliferator-activated receptor gamma (PPARγ). These changes inhibit insulin signaling and promote lipid accumulation in adipocytes (43), a finding corroborated by later work (132). Additionally, RBP4 binding to TLR4 on macrophages promotes NF-κB phosphorylation and NLRP3 inflammasome assembly, enhancing IL-1β expression and release. IL-1β then acts on adipocytes via its receptor to impair insulin-induced AKT phosphorylation, further exacerbating IR (45). RBP4 can also modulate crosstalk between innate and adaptive immune cells to promote IR. In RBP4-overexpressing mice, adipose-tissue macrophages secrete inflammatory factors that activate CD4+ T cells, largely through JNK-dependent activation of antigen-presenting cells (APCs). Transfer of these activated APCs into normal mice is sufficient to induce IR (51, 133).

Beyond IR, pancreatic β-cell dysfunction constitutes another major pathophysiological basis of T2DM. Clinical studies by Rong Huang et al. and others have shown an inverse correlation between circulating RBP4 levels and β-cell function (134–136). Mechanistically, basic research indicates that RBP4, via STRA6, suppresses the transcription factor insulin gene enhancer binding protein 1 (ISL-1)—a key regulator of insulin synthesis and β-cell homeostasis—thereby reducing insulin secretion (50, 137, 138).

Collectively, these findings suggest that pharmacological inhibition of RBP4 may simultaneously improve insulin sensitivity and preserve β-cell function, offering a promising novel strategy for the treatment of T2DM.

### 4.2 RBP4 and hypertension

Hypertension is a well-established risk factor and a leading cause of disability and mortality among patients with CVD (139, 140). Over the past five decades, the prevalence of hypertension in China has risen significantly, currently affecting approximately one-quarter of the adult population (141). Moreover, hypertension contributes to an estimated 43% of cardiovascular events in China (142, 143). A meta-analysis indicated that patients with preeclampsia, characterized by elevated blood pressure and proteinuria during pregnancy, exhibit higher plasma RBP4 levels compared with normotensive pregnant women, indirectly associating increased RBP4 levels with hypertension (144, 145). In individuals with prehypertension (defined as systolic blood pressure between 120 and 139 mmHg and/or diastolic blood pressure between 80 and 89 mmHg) serum RBP4 levels are elevated relative to those with normal blood pressure (146). Li et al. compared plasma RBP4 levels in 74 hypertensive patients and 46 healthy volunteers, reporting significantly higher RBP4 levels in the hypertensive group (147). This finding is consistent with studies by Majerczyk M et al. and Anna Solini et al. (31, 148). Animal studies further support this association: RBP4-knockout (RBP4-KO) mice demonstrated lower blood pressure than wild-type (WT) mice, whereas RBP4-overexpressing (RBP4-TG) mice exhibited higher blood pressure. Additionally, phosphorylation levels of endothelial nitric oxide synthase (eNOS) in arterial tissues were 150% in RBP4-KO mice and 80% in RBP4-TG mice compared with WT mice (149). Since eNOS regulates nitric oxide (NO) production, which is a key mediator of endothelium-dependent vasodilation and blood pressure control (150, 151), it can be inferred that RBP4 influences blood pressure by modulating NO production. Furthermore, research indicates that RBP4-induced IR may also be linked to hypertension (152, 153), offering another perspective on the relationship between RBP4 and hypertensive mechanisms. Collectively, these findings suggest that RBP4 could represent a potential therapeutic target for hypertension. However, further experimental studies are needed to clarify the precise role of RBP4 in the pathogenesis and progression of hypertension.

### 4.3 RBP4 and dyslipidemia

The lipids commonly assessed in clinical practice include plasma total cholesterol (TC), LDL, high-density lipoprotein (HDL), and triglycerides (TGs). Dyslipidemia is characterized by elevated levels of TC, TG, and LDL, along with reduced levels of HDL (154, 155). Elevated plasma LDL represents a significant risk factor for CVD (156), and the use of statins has been shown to effectively lower cholesterol levels and reduce CVD-related mortality worldwide (157). Studies have demonstrated a positive correlation between circulating RBP4 levels and TGs (158). Elevated RBP4 is associated not only with higher TG but also with lower HDL levels (86, 159). Research by Wessel et al. further indicated that both RBP4 and retinol are positively correlated with TG, TC, and LDL (160). Consistently, another study reported that changes in circulating RBP4 levels positively correlated with changes in LDL, small dense LDL-C, and apoB in patients (161). In summary, RBP4 is closely linked to lipid metabolism, although the specific mechanisms underlying this relationship remain unclear. Further research is needed to elucidate how RBP4 influences lipid metabolic pathways.

## 5 The potential of RBP4 as a therapeutic target for CVDs

Identifying disease-related proteins as targets for diagnosis and treatment represents a prominent research direction across numerous human diseases. Consequently, RBP4 holds promise as a potential target for the early diagnosis and intervention of CVDs. Its application could help shift the window for prevention and treatment forward, thereby contributing to improved public health outcomes. Numerous RBP4 antagonists have demonstrated safety and efficacy in preclinical studies. Fenretinide, a synthetic retinoid, binds to RBP4 and inhibits its interaction with TTR, promoting renal clearance of RBP4 (162, 163). Furthermore, non-retinoid ligands such as A1120 and BPN-14136 have also been shown to reduce serum RBP4 levels in animal models (164–168). Given their efficacy and safety profiles, non-retinoid RBP4 antagonists may emerge as viable therapeutic candidates. Notably, two non-retinoid antagonists, tinlarebant (BPN-14967) and STG-001 (Stargazer), are currently under clinical evaluation. In the global Phase 3 DRAGON trial, tinlarebant exhibited a well-tolerated and consistent safety profile (169), while STG-001 has completed Phase II clinical trials (170).

Beyond receptor antagonism, directly inhibiting RBP4 gene expression offers another viable strategy for reducing its serum levels. Research indicates that several antidiabetic agents, including pioglitazone (171, 172), rosiglitazone (173), and sitagliptin (174, 175), can downregulate RBP4 expression. Additionally, the lipid-lowering drug fenofibrate has been reported to inhibit RBP4 transcription in adipocytes. In a clinical study involving 15 non-diabetic patients with IR, daily administration of 200 mg fenofibrate for eight weeks resulted in a reduction of serum RBP4 levels by approximately 29.8% (176). With growing public interest in health maintenance, dietary and nutraceutical approaches have gained considerable attention. Anthocyanins, a class of water-soluble natural pigments widely present in plants, have been shown to significantly suppress RBP4 expression and lower its circulating levels (177–179). Similarly, resveratrol—a natural polyphenol found in grapes, berries, and peanuts—can reduce RBP4 expression in adipocytes (180, 181). Preclinical studies further indicate that RBP4-targeting oligonucleotides effectively decrease RBP4 expression in hepatic and adipose tissues, lower circulating RBP4 levels, and improve IR and hyperglycemia in high-fat-diet animal models (182) (Table 4).

**Table 4:** Overview of therapeutic strategies targeting RBP4.

Agent	Type	Mechanism	Current Stage	Pros	Cons	References
Fenretinide	Synthesized retinol	Promote the clearance of RBP4 from the kidneys	Phase II clinical trial	Unknown	Low bioavailability; causes night blindness	(162, 163)
A1120	Nonretinol ligands	Reduce serum RBP4 levels	Animal experiment	May avoid the retinoids-associated side effects, such as nyctalopia and delayed dark adaptation	Poor human liver microsomal stability	(164–166, 168)
BPN-14136	Nonretinol ligands	Reduce serum RBP4 levels	Animal experiment	Without affecting the visual cycles	Unknown	(167)
Tinlarebant (BPN-14967)	Nonretinol ligands	Reduce serum RBP4 levels	Phase III clinical trials	Well-tolerated and consistent safety profile	Unknown	(169)
STG-001	Nonretinol ligands	Reduce serum RBP4 levels	Phase II clinical trials	Without affecting the visual cycles	Unknown	(170)
Antidiabetic drugs	A hypoglycemic drug	Inhibiting RBP4 gene expression	In clinical use for T2DM, but RBP4 effect is observational.	Unknown	Unknown	(171–175)
Fenofibrate	A lipid-lowering drug	Inhibit the transcription of RBP4	*In vitro* experiment and clinical study	Unknown	Unknown	(176)
Anthocyanins	Natural pigment	Reduce the expression of RBP4	Animal experiment	Unknown	Unknown	(177–179)
Resveratrol	Nutrient	Reduce the expression of RBP4	Animal experiment	Unknown	Unknown	(180, 181)
RBP4-targeting oligonucleotide	RNA oligonucleotide	Inhibit the expression of RBP4	*In vitro* and *in vivo* (animal) experiment	High genetic specificity; direct reduction of synthesis	Unknown	(182)

Collectively, these findings underscore the significant clinical potential of RBP4-targeted strategies for CVD treatment. The translation of such approaches into clinical practice appears promising. However, further investigation is necessary to fully understand the implications of RBP4 inhibition on retinoid metabolism and long-term physiological outcomes.

## 6 Conclusion

**Figure 2:**
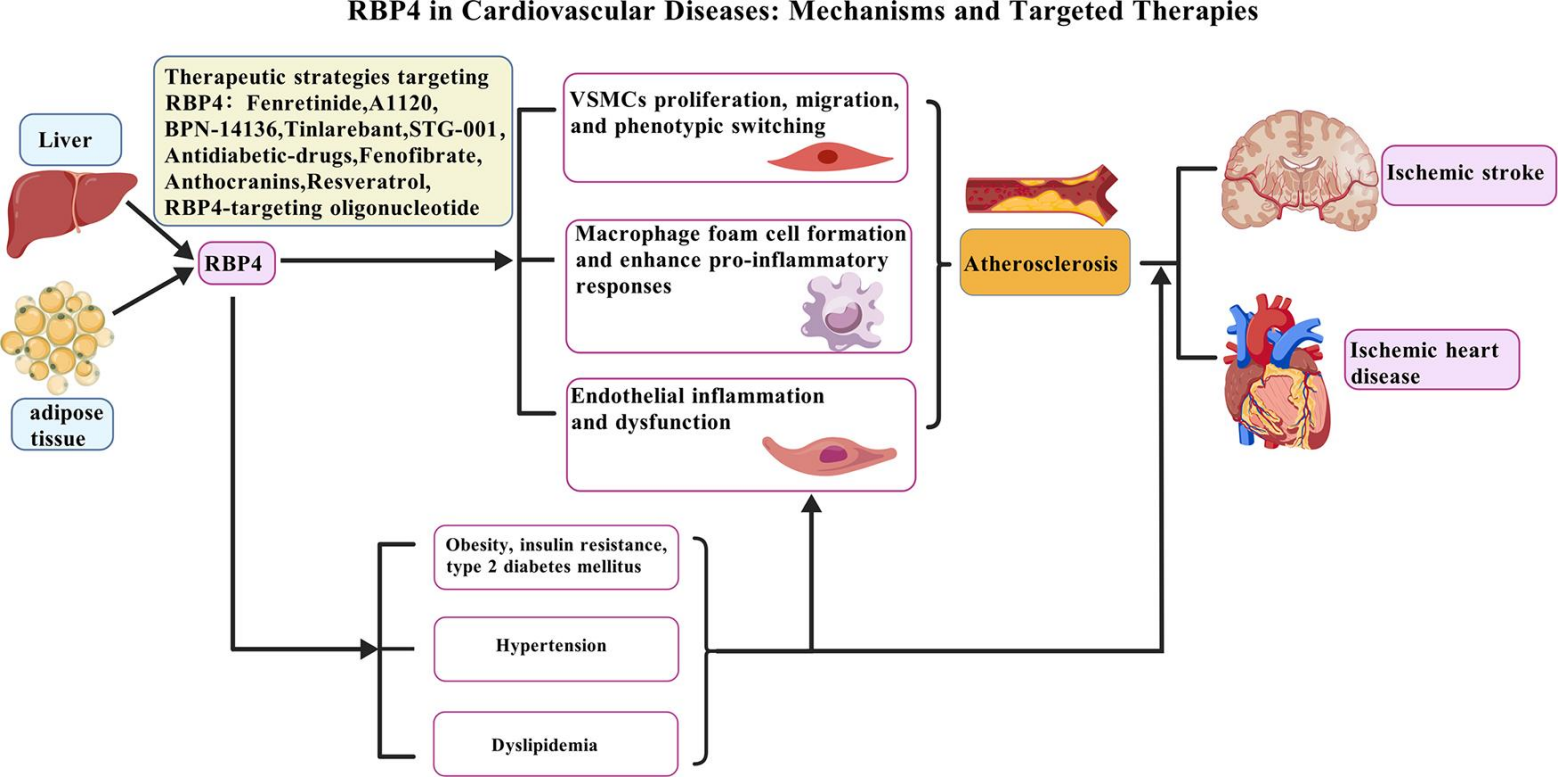
Pathogenic role of RBP4 in CVDs and potential therapeutic strategies. The increase of RBP4 level is not only related to CVDs risk factors such as obesity, insulin resistance, T2DM, hypertension and dyslipidemia, but also directly leads to the occurrence and development of atherosclerosis by acting on multiple cell types: (1) promoting VSMCs proliferation, migration, and phenotypic switching; (2) enhancing macrophage foam cell formation and pro-inflammatory responses; and (3) inducing endothelial inflammation and dysfunction. These mechanisms collectively drive the progression of CVDs, such as ischemic heart disease and ischemic stroke. Various therapeutic agents under investigation target RBP4, such as Fenretinide, BPN-14136, Tiniarebant, an RBP4-targeting oligonucleotide, as well as metabolic regulators including Resveratrol and Fenofibrate. Created with BioGDP.com.

In summary, elevated RBP4 levels are significantly associated with an increased risk of major CVDs, such as myocardial infarction and stroke. This relationship appears to operate through and interact with key cardiometabolic risk factors, particularly IR, T2DM, hypertension, and dyslipidemia [Figure 2; created with BioGDP.com (183)]. Available evidence indicates that RBP4 may function not only as a biomarker but also as an active contributor to cardiovascular pathology. Further studies are warranted to fully clarify its underlying molecular mechanisms, which may ultimately inform the development of novel preventive and therapeutic strategies for cardiovascular disorders.
